# Yeast culture improves growth, antioxidant status, immunity, and gut microbiota homeostasis in preweaning Holstein calves

**DOI:** 10.3389/fvets.2025.1670912

**Published:** 2025-09-08

**Authors:** Xueqiang Li, Xiaolin Yang, Shixiong Liu, Xi Liang, Hui Chen, Dacheng Liu

**Affiliations:** ^1^College of Veterinary Medicine, Inner Mongolia Agricultural University, Hohhot, China; ^2^Key Laboratory of Clinical Diagnosis and Treatment of Animal Diseases, Ministry of Agriculture, Hohhot, China; ^3^National Center of Technology Innovation for Dairy, Hohhot, China

**Keywords:** yeast culture, diarrhea rate, growth performance, antioxidant capacity, immune function, intestinal microbiota structure

## Abstract

**Introduction:**

Calves in the lactation period exhibit limited disease resistance and stress tolerance, making them particularly vulnerable to health challenges such as diarrhoea. Yeast culture (YC) supplementation has emerged as a promising strategy to enhance health and growth in young ruminants. This study aimed to investigate the effects of YC supplementation on growth performance, antioxidant capacity, immune function, and intestinal microbiota composition in lactating Holstein calves.

**Methods:**

A total of 40 lactating Holstein calves were randomly assigned to either a control group or a YC-supplemented group, with the feeding trial lasting 60 days. Growth performance parameters were recorded, serum antioxidant and immune markers were evaluated, and gut microbial diversity and composition were analysed using metagenomic sequencing. Furthermore, correlations between microbial taxa and serum markers were assessed.

**Results:**

The results showed that YC supplementation significantly increased average daily gain (ADG) and feed intake, and reduced the incidence of diarrhoea (*p* < 0.05). Serum levels of total antioxidant capacity (T-AOC), total superoxide dismutase (T-SOD), and glutathione peroxidase (GSH-Px) were significantly elevated, while malondialdehyde (MDA) content was significantly decreased (*p* < 0.05), indicating improved antioxidant status. Immunoglobulin and cytokine levels were also significantly higher in the YC group (*p* < 0.05). Metagenomic analysis revealed a significant increase in the Chao index and a trend toward higher Shannon diversity in the YC group. YC supplementation notably increased the relative abundance of beneficial bacteria such as *Phocaeicola plebeius*, *Ruminococcus* sp., *Segatella copri*, and *Candidatus Scatovivens faecipullorum*, while reducing potentially pathogenic bacteria like *Candidatus Cryptobacteroides* sp. and *Dorea* sp. Correlation analysis showed that T-AOC was positively associated with *P. plebeius* and *S. copri*, while MDA was positively correlated with *Candidatus Cryptobacteroides* sp. and negatively correlated with *Ruminococcus* sp. and other beneficial taxa. Similarly, several immune markers exhibited positive correlations with beneficial bacteria and negative correlations with harmful bacteria. Functional pathway analysis suggests that YC may enhance immune responses and antioxidant capacity through activation of the T cell receptor and B cell receptor signalling pathways.

**Discussion:**

In conclusion, YC supplementation improved growth performance, enhanced antioxidant and immune functions, and favourably modulated gut microbiota in lactating Holstein calves. These changes collectively contributed to reduced diarrhoea incidence and improved overall health, highlighting yeast culture as a valuable nutritional strategy for calf health management.

## 1 Introduction

In recent years, the level of dairy farming in China has steadily improved. However, during the suckling period, calves have immature gastrointestinal and immune systems, making them highly susceptible to environmental stressors and pathogenic infections. Among these, diarrhoea remains the most prevalent issue, with incidence rates reaching up to 60%. This not only hinders healthy growth and development but also negatively impacts future production performance, posing a major challenge in modern dairy farming and significantly reducing farm profitability. Therefore, enhancing immune function, maintaining gastrointestinal microecological balance, and reducing the incidence of diarrhoea during the suckling phase have become critical areas of focus in calf health management (1, 2).

Antibiotics are commonly used to prevent and treat calf diarrhoea and to promote growth. However, the drawbacks of long-term or inappropriate antibiotic use have become increasingly apparent, including the emergence of antimicrobial resistance, disruption of the gut microbial ecosystem, and concerns over drug residues in animal-derived products (3, 4). Against this backdrop, yeast culture (YC) has gained widespread application in ruminant production. Studies have demonstrated that YC can regulate rumen function, improve nutrient digestion and absorption, optimize gut microbiota structure, maintain microbial homeostasis, and enhance both immune function and antioxidant capacity (5–7).

Despite its broad application, the effectiveness and consistency of YC remain variable under commercial conditions. Differences in yeast strains, fermentation substrates, production conditions, and processing technologies across manufacturers can result in substantial variability in the composition and activity of functional components (8). Furthermore, animal species, rearing conditions, and dietary formulations can also influence YC efficacy (6), limiting its precision and large-scale application.

The YC preparation used in this study was specifically developed by our research team for ruminant animals. By selecting optimized yeast strains, fermentation substrates, and refining production processes, we ensured high functional component content, enhanced bioavailability, and improved bioactivity (9–11). Previous studies by our team in beef cattle and sheep demonstrated that this YC significantly increased average daily gain, improved antioxidant and immune function, and enhanced economic return (7, 12). Building on this foundation, the present study investigated the effects of this YC formulation on growth performance, incidence of diarrhoea, antioxidant status, immune function, and intestinal microbiota composition in lactating Holstein calves. The objective is to provide a scientific basis and technical support for the broader application of this YC product in calf rearing practices.

## 2 Materials and methods

### 2.1 Preparation of yeast culture

The XR4 strain of *Saccharomyces cerevisiae* and the BC strain of *Kluyveromyces marxianus*, both proprietary strains with independent intellectual property rights, were selected for this study. The two strains were mixed at a 1:1 ratio to prepare a fermentation culture containing 1.5 × 10^8^ CFU/g. This culture was inoculated at a rate of 10% (w/w) based on the mass of the solid substrate. Water was added to adjust the initial moisture content of the substrate to 38–40%. The formulation of the solid fermentation substrate is detailed in Supplementary Appendix 1 (9, 10).

Solid-state fermentation was carried out in a designated fermentation workshop, with the substrate stacked to a height of 60–65 cm. The temperature of the substrate was recorded every 3 h during the fermentation process. When fermentation reached 24 h and the temperature exceeded 40°C, the substrate was turned once to ensure uniform fermentation. After turning, the pile was left to ferment for the remaining period, completing a total fermentation time of 72 h. Upon completion, the fermented substrate was subjected to low-temperature drying, followed by grinding and packaging, resulting in the final yeast culture (YC) product. The nutritional composition of the YC is presented in Supplementary Appendix 2 (11).

### 2.2 Animals, diets, and experimental design

This study was conducted at Ainiu Livestock Co., Ltd. (Suihua City, Heilongjiang Province, China). A total of 40 healthy Holstein heifer calves, 3 days of age, with similar body weights (38.53 ± 1.82 kg) from the same cohort were selected and randomly assigned to either a control group or a yeast culture (YC) group (*n* = 20 per group). Each calf was housed individually in a 2.0 × 1.8 m pen. The control group received regular milk and starter feed along with a daily supplement of 30 g of fermented substrate. In contrast, the YC group received regular milk and starter feed supplemented with 30 g of yeast culture per day. The trial lasted for 67 days, including a 7-day adaptation period followed by a 60-day formal experimental period. Milk was fed twice daily at 07:30 and 16:30, according to the schedule detailed in Table 1. Starter feed was introduced from day 7 of age, offered in small and frequent portions to ensure freshness and intake. Calves had ad libitum access to starter feed and clean water throughout the experimental period. All pens were cleaned and disinfected daily according to standard farm procedures.

**Table 1:** Feeding amount of regular milk for calves (L/time).

Days of age	Feeding amount
4 ~ 10	2.0
11 ~ 20	4.0
21 ~ 25	5.0
26 ~ 60	6.0
61 ~ 65	4.5
66	4.0
67	3.5
68	3.0
69	2.5
70	2.0

The starter feed was formulated based on the Nutrient Requirements of Dairy Cattle (NRC, 2001) (13). The composition and nutrient levels are shown in Table 2. Dry matter (DM) was determined using AOAC Method 934.01 (2005) by oven-drying at 105°C to constant weight. Crude protein (CP) was analyzed via the Kjeldahl method using AOAC Method 990.03 (2005) (14). Neutral detergent fiber (NDF) and acid detergent fiber (ADF) contents were determined according to the procedures described by Van Soest et al. (15).

**Table 2:** Composition and nutrient levels of starter (%, DM basis).

Ingredients	Content
Corn grain	57.7
Soybean meal	19.3
Cottonseed meal	15.0
Wheat bran	3.0
Premix^1^	5.0
Total	100.0
Nutrient levels^2^	
NE_mf_ /(MJ/kg)	5.61
DM	91.6
CP	20.03
ADF	14.07
NDF	24.47
Ca	0.81
P	0.51

^1^The premix provided the following per kg of pellet diels: VA 250000 IU, VD 60000 IU, VE 1000 lU, Fe 80 mg, Cu 12 mg, Zn 80 mg, Mn 70 mg, Se 0.40 mg, I 1 mg, Co 0.80 mg. ^2^NE_mf_ was calculated by reference to NASEM (2016), while the others were measured values.

All experimental procedures were approved by the Animal Welfare and Ethics Committee of Inner Mongolia Agricultural University (Approval No. NND2023123) and were conducted in accordance with the guidelines for the care and use of laboratory animals (SYXK 2022–0031), as established by the National Research Council of China.

### 2.3 Sample collection

#### 2.3.1 Collection of serum samples

Blood samples (10 mL per calf) were collected from the jugular vein into anticoagulant tubes on Days 1, 30, and 60 of the formal trial period, prior to the morning feeding. Samples were centrifuged at 3,000 rpm for 10 min, and the resulting serum was aliquoted into 2 mL sterile, enzyme-free cryogenic tubes, labeled, and stored at −80°C for subsequent analysis. The following parameters were determined from serum samples: immunoglobulin G (IgG), immunoglobulin A (IgA), immunoglobulin M (IgM), lysozyme (LZM), interleukin-2 (IL-2), interleukin-6 (IL-6), interleukin-10 (IL-10), tumor necrosis factor-*α* (TNF-α), malondialdehyde (MDA), acid phosphatase (ACP), total superoxide dismutase (T-SOD), glutathione peroxidase (GSH-Px), and total antioxidant capacity (T-AOC). Additionally, 10 mL of blood was collected into anticoagulant tubes and stored at 4°C for transportation to the laboratory, where lymphocyte proliferation capacity (LPC) was measured.

#### 2.3.2 Collection of fecal samples

On the designated sampling day, fresh fecal samples were collected from six randomly selected, clinically healthy calves in each group. Using sterile disposable gloves, approximately 10 g of feces was obtained directly from the rectum to minimize environmental contamination. Samples were placed in sterile centrifuge tubes, immediately frozen in liquid nitrogen, and then transferred to a − 80°C freezer for storage prior to DNA extraction and metagenomic sequencing analysis.

### 2.4 Index measurement and methods

#### 2.4.1 Measurement of growth performance indexes

On Days 1, 30, and 60 of the formal trial period, calves were weighed before the morning feeding, following an overnight fast. Daily feed intake and refusals were recorded throughout the trial, and the average daily gain (ADG) and average daily feed intake (ADFI) were calculated accordingly.

The relevant calculation formulas are as follows:


$$\begin{array}{l} \mathrm{ADFI}\;\left(g/d\right)=\\ \left[\mathrm{total feed amounts}\;\left(g\right)-\mathrm{total residue amounts}\;\left(g\right)\right]/\mathrm{trial days}. \end{array}$$

$$\begin{array}{l} \mathrm{ADG}\;\left(\mathrm{kg}/d\right)=\left(\mathrm{final body weights}-\mathrm{initial body weights}\right)\\ \;\;\;\;\;\;\;\;\;\;\;\;\;\;\;\;\;\;\;\;\;\;\;\;\;\;\;\;\;\;\;\;\;\;\;\;\;/\mathrm{trial days}. \end{array}$$

#### 2.4.2 Faecal scoring and Diarrhoea rate

Faecal consistency was assessed daily at 07:00 and 18:00 during the trial period using the scoring method described by Larson et al. (16). (refer to Table 3 for scoring criteria). A score ≥3 was considered indicative of diarrhoea. The number of diarrhoeic events was recorded for each calf, with a maximum of one episode per calf per day. The diarrhoea rate was calculated using the following formula:

**Table 3:** Scoring standard of calf feces.

Appearance	Score standard	Score
Normal	Well-formed and not loose	1
Formed	Soft but formable	2
Unformed	Loose, paste-like	3
Unformed	Watery, easy to spread	4
Unformed	Watery, separated fecal matter, splashing	5

Diarrhoea rate (%) = [(Number of diarrhoeic calves × Number of diarrhoea days) / (Total number of calves × Total number of trial days)] × 100.

#### 2.4.3 Determination of antioxidant capacity indexes

The antioxidant indicators, including total antioxidant capacity (T-AOC), total superoxide dismutase (T-SOD), glutathione peroxidase (GSH-Px), and malondialdehyde (MDA), were measured in serum samples using commercial assay kits (Table 4). All kits were obtained from Nanjing Jiancheng Bioengineering Institute (Nanjing, China), and assays were performed strictly according to the manufacturer’s instructions.

**Table 4:** Serum antioxidant indicator test kits and their product numbers and measurement methods.

Items	Kit names	method	Product numbers
T-AOC	Total antioxidant capacity assay kit	ABTS	A015-2
T-SOD	Superoxide dismutase (SOD) assay kit	WST-1	A001-3
GSH-Px	Glutathione peroxidase (GSH-Px) assay kit	Colorimetric method	A005-1
MDA	Malondialdehyde (MDA) assay kit	TBA	A003-1

#### 2.4.4 Determination of immune function indexes

Serum levels of immunoglobulin G (IgG), immunoglobulin A (IgA), immunoglobulin M (IgM), lysozyme (LZM), interleukin-2 (IL-2), interleukin-6 (IL-6), interleukin-10 (IL-10), tumor necrosis factor-*α* (TNF-α), acid phosphatase (ACP), and lymphocyte proliferation capacity (LPC) were determined using commercially available kits (Table 5) from Nanjing Jiancheng Bioengineering Institute. All procedures followed the protocols provided by the manufacturer.

**Table 5:** Serum immunological indicator test kit and its product number, testing method.

Items	Kit names	method	Product numbers
IgG	Immunoglobulin G Assay Kit	ELISA	H106-1-1
IgA	Immunoglobulin A Assay Kit	ELISA	H108-1-2
IgM	Immunoglobulin M Assay Kit	ELISA	H109-1-1
IL-2	Interleukin −2 Assay Kit	ELISA	H003-1-2
IL-6	Interleukin −6	ELISA	H007-1-2
IL-10	Interleukin −10 Assay Kit	ELISA	H009-1-2
TNF-α	Tumor Necrosis Factor-*α* Assay Kit	ELISA	H052-1-2
LZM	Lysozyme assay kit	Turbidimetric inhibition assay	A050-1-1
ACP	Acid phosphatase assay kit	Spectrophotometry	A060-1-1
LPC	MTT cell proliferation and cytotoxicity assay kit	MTT	G020-1-1

#### 2.4.5 Metagenomic sequencing analysis

Total DNA was extracted from fecal samples using the E.Z.N.A.® Soil DNA Kit (Omega Bio-tek, Norcross, GA, USA) following the manufacturer’s instructions. DNA integrity was assessed via 1% agarose gel electrophoresis, while DNA concentration and purity were determined using a spectrophotometer. Genomic DNA was then sheared to approximately 350 bp fragments using a Covaris M220 ultrasonicator (Covaris, USA), and paired-end libraries were constructed using the NEXTFLEX® Rapid DNA-Seq Kit (Bioo Scientific, USA).

Metagenomic sequencing was performed on the Illumina NovaSeq™ X Plus platform (Illumina, USA), with sequencing services provided by Shanghai Meiji Biotechnology Co., Ltd. Raw reads obtained from sequencing were first aligned to the host reference genome using BWA (v0.7.17) (17) to remove host-derived sequences. The remaining high-quality reads were *de novo* assembled using MEGAHIT (v1.1.2) (18), and contigs of ≥300 bp in length were retained for downstream analysis.

Open reading frames (ORFs) were predicted from the assembled contigs using Prodigal (v2.6.3), and coding sequences ≥100 bp were translated into corresponding amino acid sequences. All predicted protein sequences were clustered using CD-HIT (v4.7) (19) with a 90% sequence identity and 90% coverage threshold to construct a non-redundant gene catalog. The longest sequence in each cluster was selected as the representative sequence.

High-quality reads from each sample were aligned to the non-redundant gene set using SOAPaligner (v2.21) (20) with a 95% identity threshold to calculate gene abundance. Taxonomic annotation was performed by aligning the amino acid sequences of the non-redundant gene set to the NCBI NR database using DIAMOND (v2.0.13) (21) with the BLASTP algorithm (e-value < 1e-5). Species-level relative abundance was calculated by summing the abundance of genes annotated to the same species.

### 2.5 Statistical analysis

All data were initially organized and processed using Microsoft Excel 2010. Statistical analyses for growth performance, antioxidant capacity, and immune function indicators were performed using the independent samples t-test in IBM SPSS Statistics 22.0 (IBM Corp., Armonk, NY, USA). Results are presented as mean ± standard deviation (SD). Differences were considered statistically significant at *p* < 0.05 and highly significant at *p* < 0.01.

## 3 Results

### 3.1 Effects of yeast culture on growth performance of calves

As shown in Table 6, the average daily feed intake (ADFI) over the entire trial period was significantly higher in the YC group (200.3 g/day) than in the control group (172.6 g/day), representing a 16.05% increase (*p* < 0.01). From Days 1 to 30 and Days 31 to 60, ADFI in the YC group increased by 24.78 and 14.60%, respectively, compared with the control group; however, these differences were not statistically significant (*p* > 0.05). There was no significant difference in initial body weight between the two groups on Day 1 (*p* > 0.05). On Day 30, the body weight of YC-fed calves was slightly higher than that of the control group, but the difference remained nonsignificant (*p* > 0.05). By Day 60, however, the body weight of the YC group increased significantly to 93.50 kg, which was 6.25% higher than the control group (*p* < 0.01). Over the full 60-day experimental period, the average daily gain (ADG) of the YC group reached 0.92 kg/day, significantly greater than 0.82 kg/day in the control group (*p* < 0.01), corresponding to a 12.20% increase.

**Table 6:** Effects of YC on growth performance of calves.

Items	Time points	Control group	YC group	*p*-value
ADFI (g/d)	Day 1 ~ 30	48.11 ± 9.26^a^	60.03 ± 8.47^b^	0.042
	Day 31 ~ 60	297.2 ± 11.87^A^	340.6 ± 19.69^B^	<0.01
	Day 1 ~ 60	172.6 ± 4.05^A^	200.3 ± 12.17^B^	<0.01
Body weight/kg	Day 1	38.73 ± 1.20	38.47 ± 1.40	0.731
	Day 30	59.83 ± 2.40	61.83 ± 2.64	0.200
	Day 60	88.00 ± 2.61^A^	93.50 ± 3.02^B^	<0.01
ADG(Kg/d)	Day 1 ~ 30	0.70 ± 0.07	0.78 ± 0.06	0.083
	Day 31 ~ 60	0.94 ± 0.08	1.06 ± 0.13	0.093
	Day 1 ~ 60	0.82 ± 0.04^A^	0.92 ± 0.06^B^	<0.01

In the same row, values with no letter or the same letter superscripts mean no significant difference (*P* > 0.05), while with different small letter superscripts mean significant difference (*P* < 0.05), and with different capital letter superscripts mean significant difference (*p* < 0.01). The same as below. The value is the mean ± SD, *n* = 20.

ADG, average daily gain.

ADFI; average daily feed intake.

These findings suggest that supplementation with yeast culture significantly improves both feed intake and growth rate in calves.

### 3.2 Effects of yeast culture on faecal score and diarrhoea rate

As shown in Table 7, the average faecal score and diarrhoea rate of calves in the YC group throughout the 60-day trial were significantly lower than those in the control group (*p* < 0.05), with values of 1.78 and 3.42%, respectively. Specifically, from Days 1 to 30, the faecal score and diarrhoea rate in the YC group decreased by 0.21 and 8.33%, respectively (*p* < 0.01). From Days 31 to 60, the diarrhoea rate was 2.17% lower in the YC group (*p* < 0.05).

**Table 7:** Effects of YC on faecal score and diarrhoea rate.

Items	Time points	Control group	YC group	*P*-value
Fecal score/score	Day 1 ~ 30	2.31 ± 0.06^A^	2.10 ± 0.05^B^	<0.01
	Day 31 ~ 60	1.62 ± 0.12	1.45 ± 0.22	0.224
	Day 1 ~ 60	1.97 ± 0.07^a^	1.78 ± 0.12^b^	0.036
Diarrhea rate/%	Day 1 ~ 30	13.33 ± 2.72^A^	5.00 ± 1.59^B^	<0.01
	Day 31 ~ 60	4.0 ± 1.09^a^	1.83 ± 0.64^b^	0.014
	Day 1 ~ 60	8.67 ± 1.91^A^	3.42 ± 1.00^B^	<0.01

In the same row, values with no letter or the same letter superscripts mean no significant difference (*P*>0.05), while with different small letter superscripts mean significant difference (*P*<0.05), and with different capital letter superscripts mean significant difference (*P* < 0.01). The same as below. The value is the mean ± SD, *n* = 20.

These results indicate that yeast culture supplementation helps improve intestinal health and reduce the incidence of diarrhoea in calves.

### 3.3 Effects of yeast culture on antioxidant capacity in calves

As shown in Table 8, there were no significant differences in serum antioxidant indices between the two groups at the beginning of the experiment (Day 1) (*p* > 0.05). On Day 30, the YC group exhibited significantly higher levels of T-SOD activity, T-AOC, and GSH-Px activity compared to the control group, with increases of 1.34 U/mL, 0.11 mmol/L, and 35.2 U/mL, respectively (*p* < 0.05). No significant difference was observed in MDA levels at this time point (*p* > 0.05).

**Table 8:** Effects of YC on antioxidant capacity in calves.

Items	Time points	Control group	YC group	*P*-value
T-SOD/(U/mL)	Day 1	17.05 ± 1.68	16.83 ± 2.14	0.861
	Day 30	17.24 ± 0.63^a^	18.58 ± 1.06^b^	0.041
	Day 60	18.06 ± 0.68^a^	19.47 ± 1.06^b^	0.038
T-AOC/(mmol/L)	Day 1	0.48 ± 0.07	0.47 ± 0.09	0.850
	Day 30	0.52 ± 0.08^a^	0.63 ± 0.05^b^	0.041
	Day 60	0.61 ± 0.09^a^	0.75 ± 0.09^b^	0.043
GSH-Px/(U/mL)	Day 1	49.38 ± 3.95	49.53 ± 4.31	0.955
	Day 30	53.44 ± 11.21^A^	88.64 ± 13.71^B^	0.002
	Day 60	127.1 ± 12.94^a^	144.6 ± 15.19^b^	0.046
MDA/(nmol/mL)	Day 1	2.83 ± 0.52	2.87 ± 0.49	0.892
	Day 30	3.52 ± 0.78	3.47 ± 0.68	0.924
	Day 60	4.22 ± 0.51^A^	2.51 ± 0.33^B^	<0.01

In the same row, values with no letter or the same letter superscripts mean no significant difference (*P* > 0.05), while with different small letter superscripts mean significant difference (*P* < 0.05), and with different capital letter superscripts mean significant difference (*P* < 0.01). The same as below. The value is the mean ± SD.

By Day 60, the YC group showed significant improvements in serum antioxidant capacity: T-SOD and GSH-Px activities and T-AOC levels increased by 7.81, 13.77, and 22.95%, respectively (*p* < 0.05), while MDA content decreased by 40.52% (*p* < 0.01).

These findings indicate that prolonged supplementation with yeast culture significantly enhances the antioxidant defense system in calves.

### 3.4 Effects of yeast culture on immune function in calves

As shown in Table 9, there were no significant differences in serum immunoglobulin levels (IgG, IgA, IgM) between the two groups on Day 1 (*p* > 0.05). By Day 30, serum IgG concentrations in the YC group were significantly higher than in the control group (*p* < 0.05). On Day 60, IgG, IgA, and IgM levels in the YC group were increased by 12.10, 13.69, and 13.19%, respectively (*p* < 0.05).

**Table 9:** Effects of YC on immune function in calves.

Items	Time points	Control group	YC group	*P*-value
IgG/(μg/ml)	Day 1	832.4 ± 46.89	821.6 ± 24.29	0.627
	Day 30	894.7 ± 62.86^a^	972.8 ± 53.90^b^	0.044
	Day 60	996.4 ± 52.76^a^	1,117 ± 76.5^b^	0.010
IgA/(μg/ml)	Day 1	347.9 ± 27.26	353.6 ± 21.85	0.702
	Day 30	367.3 ± 14.10	383.7 ± 23.00	0.167
	Day 60	403.9 ± 36.62^a^	459.2 ± 37.59^b^	0.028
IgM/(μg/ml)	Day 1	103.7 ± 2.46	105.2 ± 5.59	0.561
	Day 30	110.1 ± 9.35	115.6 ± 8.45	0.313
	Day 60	113.7 ± 9.42^a^	128.7 ± 12.71^b^	0.043
LZM/(μg/ml)	Day 1	7.68 ± 0.65	7.72 ± 0.59	0.921
	Day 30	7.91 ± 0.43	8.19 ± 0.58	0.406
	Day 60	8.10 ± 0.73^A^	9.77 ± 0.67^B^	<0.01
ACP/(U/mL)	Day 1	7.50 ± 0.36	7.49 ± 0.39	0.992
	Day 30	7.63 ± 0.59^a^	9.47 ± 0.92^b^	0.036
	Day 60	8.85 ± 1.78^A^	12.38 ± 1.36^B^	<0.01
IL-2/(pg/mL)	Day 1	106.5 ± 4.50	104.0 ± 5.13	0.391
	Day 30	132.1 ± 18.20^a^	162.2 ± 19.54^b^	0.020
	Day 60	148.8 ± 7.44^A^	200.7 ± 9.37^B^	<0.01
IL-10/(pg/mL)	Day 1	23.10 ± 2.13	23.60 ± 1.62	0.652
	Day 30	23.19 ± 1.57	25.28 ± 3.80	0.243
	Day 60	26.31 ± 4.23^a^	33.69 ± 4.58^b^	0.016
TNF-α/(pg/mL)	Day 1	50.05 ± 2.39	50.71 ± 1.07	0.550
	Day 30	41.55 ± 1.75	39.13 ± 2.40	0.074
	Day 60	34.58 ± 0.96	31.50 ± 4.19	0.110
IL-6/(pg/mL)	Day 1	38.41 ± 2.03	39.42 ± 2.30	0.441
	Day 30	29.91 ± 3.08	27.44 ± 2.30	0.146
	Day 60	25.87 ± 1.17^A^	22.06 ± 1.86^B^	0.002
LPC	Day 1	1.22 ± 0.41	1.24 ± 0.37	0.941
	Day 30	1.84 ± 0.19^a^	2.32 ± 0.37^b^	0.033
	Day 60	1.47 ± 0.17^a^	1.90 ± 0.27^b^	0.016

In the same row, values with no letter or the same letter superscripts mean no significant difference (*P* > 0.05), while with different small letter superscripts mean significant difference (*P* < 0.05), and with different capital letter superscripts mean significant difference (*P* < 0.01). The same as below. The value is the mean ± SD.

There were no significant differences in other immune-related indices on Day 1. By Day 30, levels of lysozyme (LZM) and interleukin-10 (IL-10) showed an increasing trend, TNF-*α* and IL-6 levels showed an downward trend, though not statistically significant (*p* > 0.05). However, acid phosphatase (ACP) activity, interleukin-2 (IL-2), and lymphocyte proliferation capacity (LPC) were significantly elevated in the YC group by 1.84 U/mL, 30.1 pg./mL, and 0.48, respectively (*p* < 0.05). On Day 60, LZM, ACP activity, IL-2, IL-10, and LPC values in the YC group increased by 20.62, 39.89, 34.88, 28.05, and 29.25%, respectively, compared with the control group (*p* < 0.05). TNF-α and IL-6 levels decreased by 3.08 pg./mL (*p* > 0.05) and 3.81 pg./mL (*p* < 0.01), respectively.

These results demonstrate that dietary yeast culture supplementation can significantly enhance both humoral and cellular immune responses in calves.

### 3.5 Effects of yeast culture on the gut microbiota structure of lactating calves

**Figure 1:**
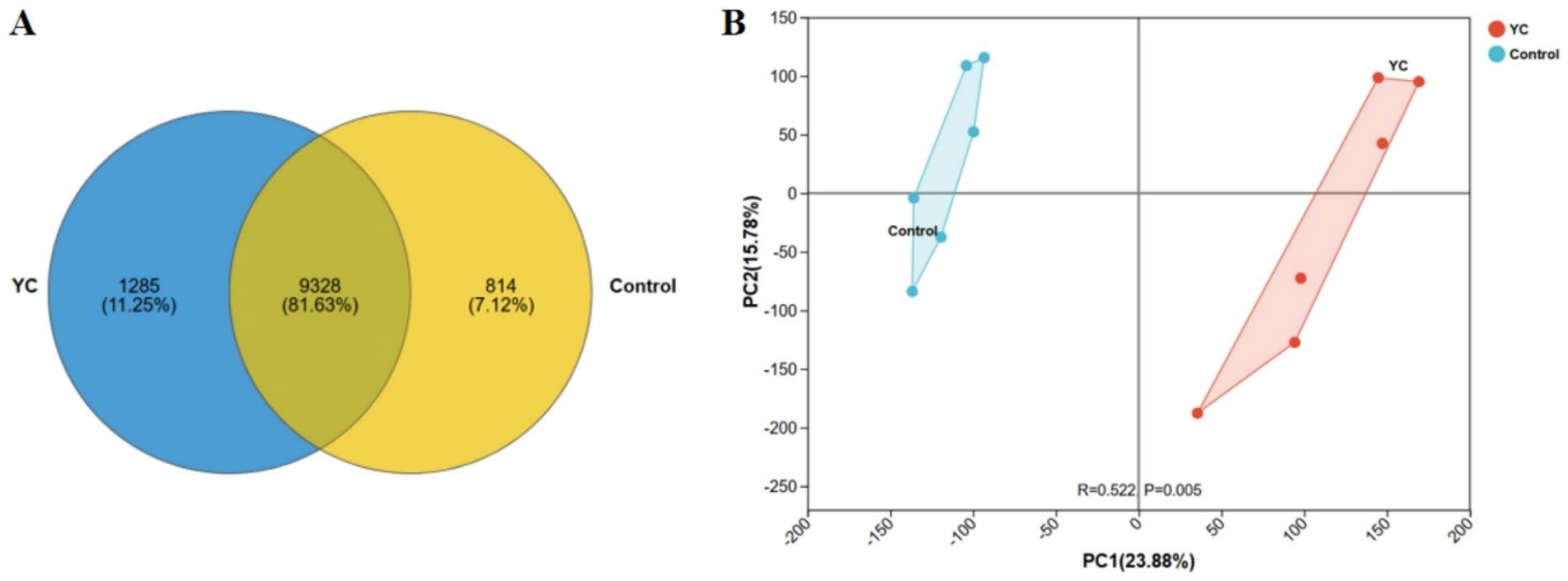
Diversity of intestinal microbiota in lactating calves. **(A)** Venn diagram highlighting the overlapping ASVs when comparing composition of gut microbiota two groups. **(B)** Principal Component Analysis (PCA) score plot showing the distribution of samples from two groups along PC1 and PC2. Percentages in parentheses indicate the explained variance. R and *p* values represent group separation and statistical significance. *n* = 6.

To evaluate the impact of yeast culture (YC) supplementation on the gut microbiota of lactating calves, metagenomic sequencing was conducted using the Illumina NovaSeq™ X Plus platform (Illumina, USA). A total of 11,427 amplicon sequence variants (ASVs) were identified across 12 samples. Among them, 9,328 ASVs were shared between groups, while 814 (7.12%) and 1,285 (11.25%) unique ASVs were observed in the control and YC groups, respectively (Figure 1A).

Alpha diversity analysis revealed that sequencing coverage exceeded 99.5% for all samples, indicating adequate sequencing depth and reliable characterization of microbial communities. As shown in Table 10, the YC group exhibited significantly higher microbial richness (Chao index) compared to the control group (*p* < 0.05). While the Shannon index showed an increasing trend and the Simpson index a decreasing trend, these differences were not statistically significant (*p* > 0.05), suggesting improved overall richness and diversity in the YC group.

**Table 10:** The effect of YC on the *α* diversity index in the sample.

Items	Control group	YC group	*P*-value
Chao	7,985 ± 216.9^a^	8,518 ± 383.9^b^	0.027
Shannon	5.31 ± 0.16	5.40 ± 0.17	0.439
Simpson	0.018 ± 0.005	0.016 ± 0.004	0.426

In the same row, values with no letter or the same letter superscripts mean no significant difference (*P* > 0.05), while with different small letter superscripts mean significant difference (*P* < 0.05), and with different capital letter superscripts mean significant difference (*P* < 0.01). The same as below. The value is the mean ± SD, *n* = 6.

Beta diversity analysis based on Euclidean distance and principal component analysis (PCA) demonstrated a clear separation between the microbial community structures of the two groups (Figure 1B). This distinction was supported by the Analysis of Similarities (ANOSIM), which confirmed a significant difference in microbial composition between groups (*p* < 0.05).

**Figure 2:**
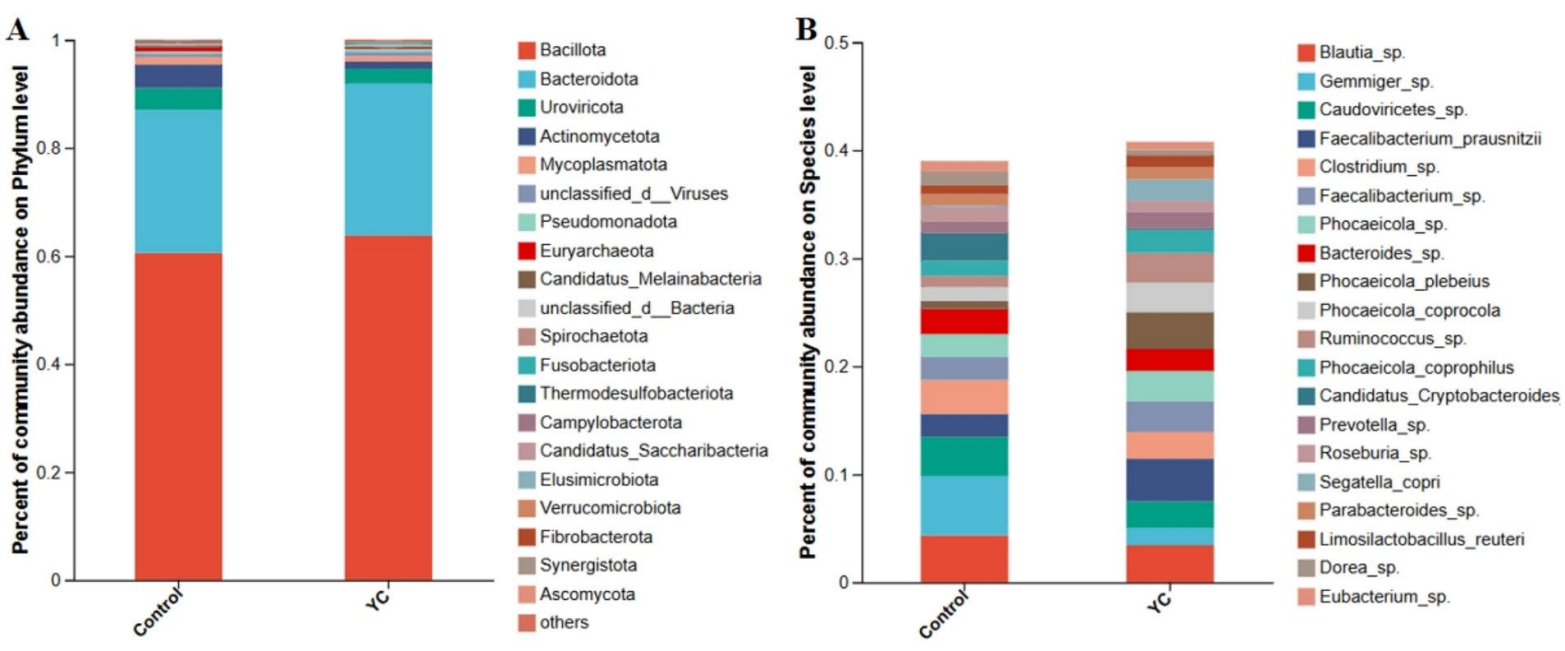
Composition of the intestinal microbiota in the lactating calves. **(A)** Composition of the gut microbiota at the phylum level. **(B)** Composition of the gut microbiota at the species level. Control = basal diet. YC = basal diet supplemented with yeast culture. *n* = 6.

**Figure 3:**
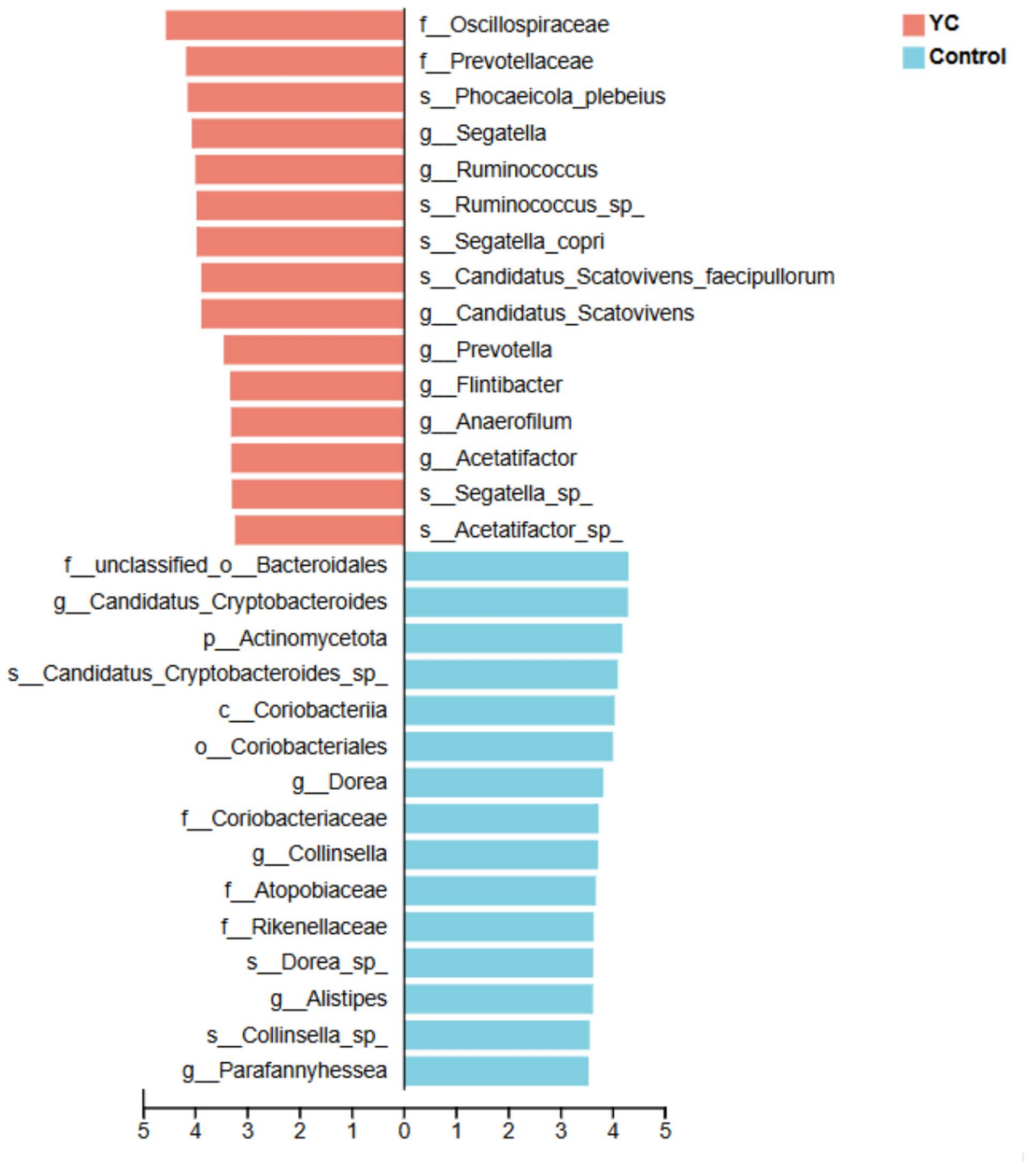
Linear Discriminant Analysis (LDA) on the species level. Bacterial taxa at the species level significantly identified by linear discriminant analysis coupled with effect size (LEfSe) using the default parameters between groups control and YC. Control, basal diet; YC, basal diet supplemented with yeast culture. *n* = 6.

At the phylum level, *Bacillota* and *Bacteroidota* were dominant in both groups (Figure 2A). At the species level, *Blautia* sp., *Gemmiger* sp., and *Caudoviricetes* sp. were the most abundant taxa (Figure 2B). Linear discriminant analysis effect size (LEfSe) identified several differentially enriched taxa (LDA score ≥ 3.0; p < 0.05). The YC group showed significantly increased relative abundances of *Phocaeicola plebeius*, *Ruminococcus* sp., *Segatella copri*, and *Candidatus Scatovivens faecipullorum*, whereas the relative abundances of *Candidatus Cryptobacteroides* sp. and *Dorea* sp. were significantly reduced (Figure 3).

These findings suggest that yeast culture supplementation alters the gut microbial structure of lactating calves, enhancing microbial richness and promoting the colonization of beneficial taxa.

### 3.6 Analyses of correlations between intestinal microbiota, antioxidant capacity, and immune function indicators

**Figure 4:**
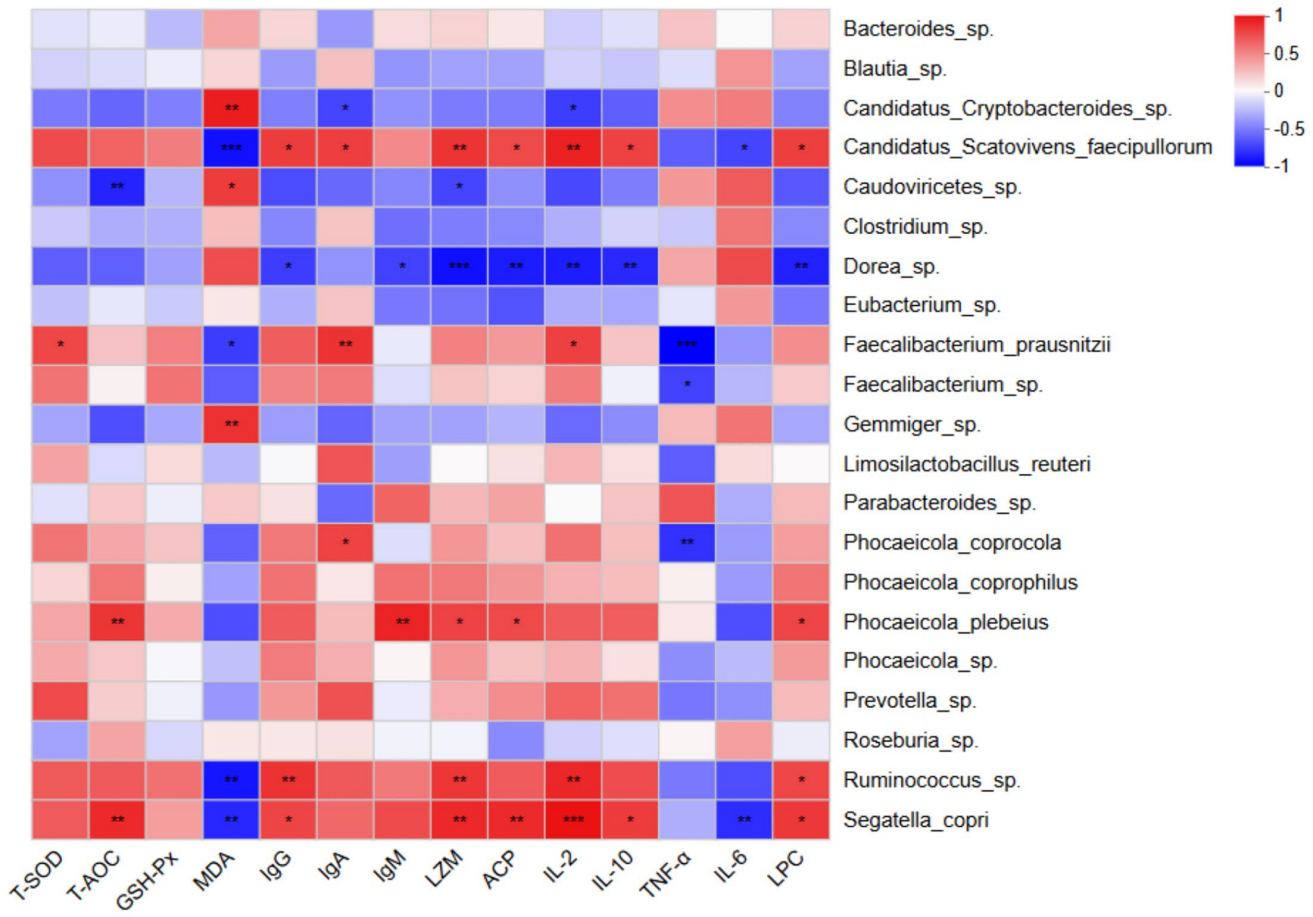
Heatmap showing Spearman correlations between intestinal bacterial species (y-axis) and serum antioxidant and immune function indicators (x-axis). Positive and negative correlations are represented by red and blue colors, respectively. The intensity of the color indicates the strength of the correlation. *p < 0.05; **p < 0.01; ***p < 0.001. *n* = 6.

Next, we investigated the correlations between serum antioxidant and immune function indicators and the dominant microbial species identified in faecal samples (Figure 4). The results demonstrated that total antioxidant capacity (T-AOC) was significantly positively correlated with the relative abundances of *Phocaeicola plebeius* and *Segatella copri* (*p* < 0.01). Conversely, malondialdehyde (MDA) levels were significantly positively correlated with the abundance of *Candidatus Cryptobacteroides* sp. (*p* < 0.01) and significantly negatively correlated with *Ruminococcus* sp., *Segatella copri*, and *Candidatus Scatovivens faecipullorum* (*p* < 0.01).

The abundances of *Phocaeicola plebeius*, *Ruminococcus* sp., *Segatella copri*, and *Candidatus Scatovivens faecipullorum* were positively correlated, to varying degrees, with serum immune function indicators including IgG, IgA, IgM, LZM, ACP, IL-2, IL-10, and LPC, while showing negative correlations with TNF-*α* and IL-6 levels. In contrast, the abundances of *Candidatus Cryptobacteroides* sp. and *Dorea* sp. were positively correlated with TNF-α and IL-6 levels, and negatively correlated, to varying degrees, with the other immune function indicators.

Collectively, these findings suggest that dietary supplementation with yeast culture during the suckling period modulates the gut microbiota composition of calves, thereby enhancing antioxidant capacity and immune function, and potentially reducing the risk of diarrhoea during early life.

### 3.7 KEGG functional enrichment analysis and functional gene differential analysis

KEGG functional enrichment analysis was conducted to assess the effects of differential microbiota on pathways associated with yeast culture supplementation. A total of 155 significantly enriched pathways were identified, including eight related to the immune system, such as the T cell receptor signalling pathway and the B cell receptor signalling pathway, which were selected for further investigation (Additional file 1).

**Figure 5:**
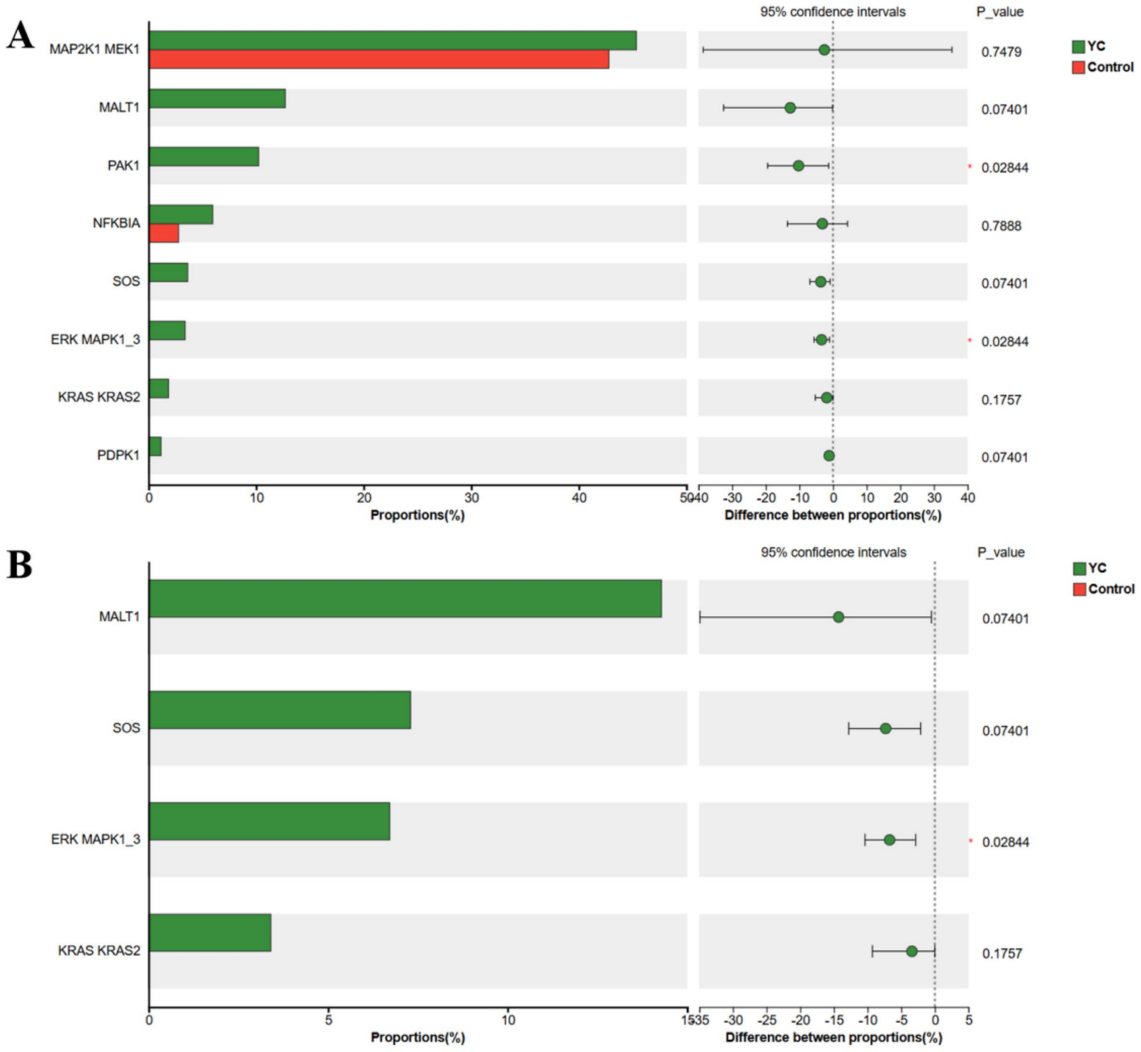
KEGG functional gene difference analysis bar chart. **(A)** Differential analysis of T cell receptor signalling pathway genes. **(B)** Differential analysis of B cell receptor signalling pathway genes. The vertical axis represents functional genes, and the horizontal axis represents the percentage of abundance. Control, basal diet; YC, basal diet supplemented with yeast culture. *p < 0.05, *n* = 6.

Subsequent differential analysis of the genes encoding functional proteins within these immune-related pathways revealed that, in the T cell receptor signalling pathway, PAK1 and ERK MAPK1_3 were significantly upregulated in the YC group. In the B cell receptor signalling pathway, ERK MAPK1_3 was also significantly upregulated in the YC group compared with the control group (Figure 5).

### 3.8 Correlation analysis between antioxidant capacity, immune function indicators, and KEGG functional genes

**Figure 6:**
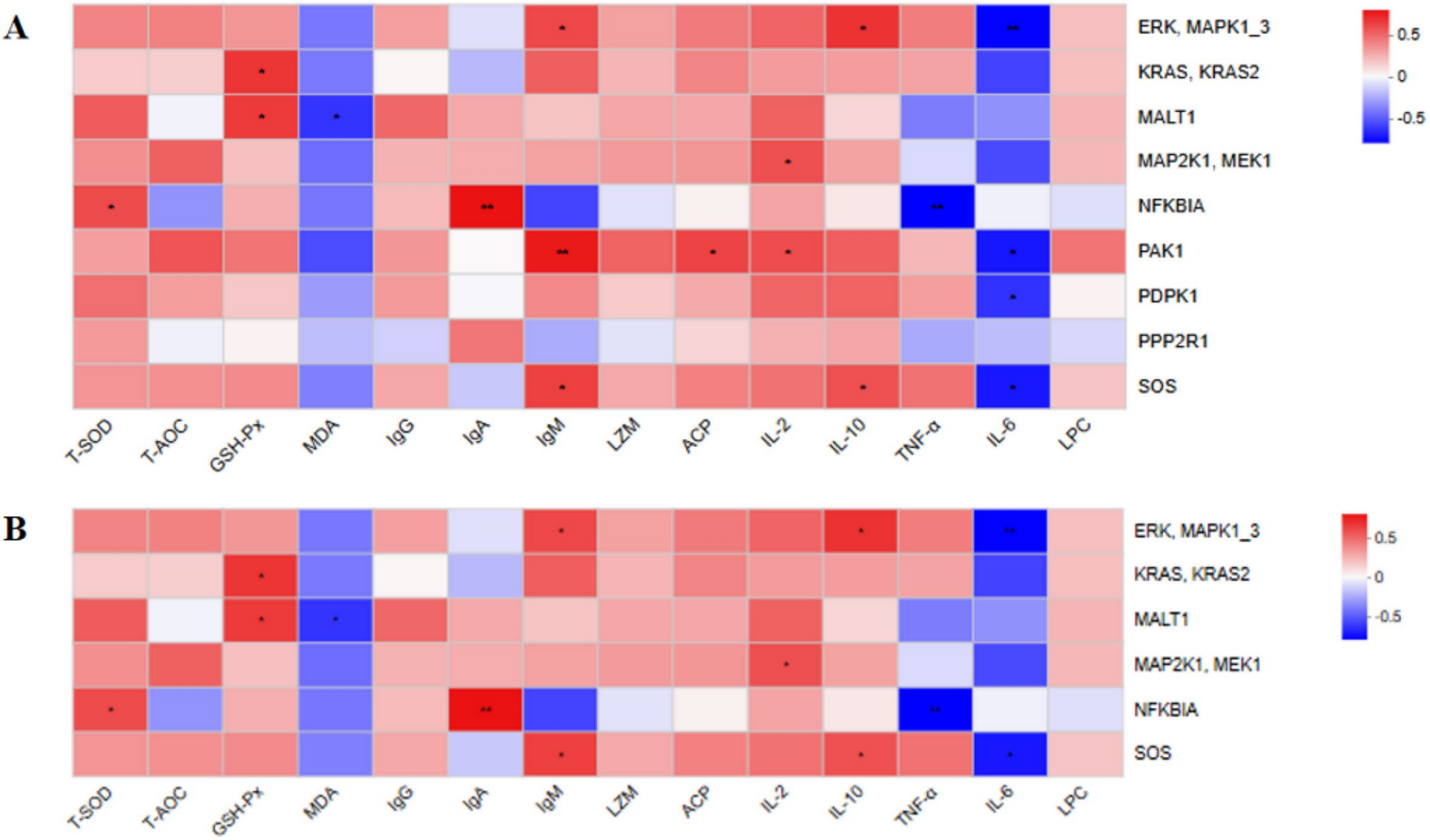
Heat map showing the correlation analysis between antioxidant capacity and immune function indicators and KEGG functional genes. Positive and negative correlations are represented by red and blue colors, respectively. The intensity of the color indicates the strength of the correlation. *p < 0.05; **p < 0.01; ***p < 0.001. *n* = 6.

We next evaluated the correlations between serum antioxidant capacity, immune function indicators, and KEGG functional genes (Figure 6). Genes enriched in the T cell receptor signaling pathway and B cell receptor signaling pathway showed positive correlations, to varying degrees, with serum antioxidant capacity indicators (T-SOD, T-AOC, and GSH-Px) and immune function indicators (IgG, IgA, IgM, LZM, ACP, IL-2, IL-10, and LPC). In contrast, these genes exhibited negative correlations, to varying degrees, with MDA, TNF-*α*, and IL-6.

These findings suggest that supplementation with YC during the suckling period may modulate immune-related signaling pathways, thereby enhancing the antioxidant capacity and immune function of calves.

## 4 Discussion

In recent years, yeast cultures (YCs) have attracted increasing attention in animal production due to their rich nutritional profile and diverse bioactive properties. They are commonly used to enhance feed digestion and nutrient absorption, modulate the intestinal microbiota, boost immune responses, improve antioxidant capacity, and mitigate physiological stress (8). Despite their widespread application in livestock systems, the effectiveness of YCs varies considerably under different production conditions. These inconsistencies largely stem from variations in yeast strains, fermentation substrates, manufacturing processes, and quality control standards, all of which can lead to differences in the type and concentration of bioactive compounds in the final product. Moreover, differences in animal species, feeding practices, and dietary compositions further influence the practical efficacy of YCs, thereby hindering their standardized application in large-scale operations (6, 22). Previous studies have indicated that YC may exert limited effects under certain conditions. For instance, under well-managed health conditions, YC supplementation did not significantly influence feed intake or body weight in calves, nor did it improve feed intake or growth performance in fattening calf trials (23, 24). To overcome these limitations, the YC used in this study was independently developed by our research team. By selecting high-performing yeast strains, optimizing fermentation substrates, and implementing precise control of fermentation parameters, we developed a preparation enriched with a broad spectrum of bioactive components, featuring improved bioavailability and enhanced product stability. This study aimed to evaluate the effects of this YC as a feed additive on key growth and health-related outcomes in suckling Holstein calves, including growth performance, incidence of diarrhoea, serum antioxidant and immune indices, and gut microbial composition.

### 4.1 Effects of yeast culture on growth performance of calves

Early feeding behavior and feed utilization efficiency in calves play a critical role in determining their growth performance and associated economic returns. Yeast cultures (YCs) are known to be rich in amino acids, vitamins, and enzymes that contribute to rumen development by increasing the absorptive surface area for nutrient digestion and uptake, optimizing the rumen microbial community, and enhancing digestive enzyme activities. These effects collectively improve feed digestion, nutrient absorption, and weight gain (5, 25, 26). In the present study, dietary supplementation with YC significantly improved average daily feed intake (ADFI) and average daily gain (ADG) in suckling calves by 16.05 and 12.20%, respectively. These findings are consistent with those reported by Salinas et al. (27) and Lesmeister et al. (28), who demonstrated that YC supplementation markedly enhances early dry matter intake and growth rates in calves, effectively shortening the growth period (27, 28). This supports the hypothesis that YC can promote feed intake and improve growth performance in young ruminants.

Our previous research further revealed that YC promotes rumen epithelial cell proliferation, accelerates papillae elongation, and facilitates rumen morphological development, which is critical for efficient nutrient absorption (29). Additionally, YC supplementation was shown to increase the relative abundance of key fiber-degrading bacterial genera—*Prevotella*, *Fibrobacter*, *Butyrivibrio*, and *Ruminococcus*—enhancing rumen fermentation efficiency and elevating the concentrations of total volatile fatty acids (VFAs), including acetate, propionate, and butyrate. Moreover, the expression of genes associated with nutrient transport and absorption in rumen epithelial cells—such as *MCT1*, *NHE1*, *NHE3*, *PAT1*, and *vH^+^-ATPase*—was significantly upregulated (30). Together, these results suggest that YC enhances rumen development and functionality, thereby promoting nutrient digestion and absorption and ultimately contributing to improved growth performance in calves.

### 4.2 Effects of yeast culture on antioxidant capacity, immune function, and diarrhoea rate in lactating calves

The lactation period represents a critical window for calf growth and development. However, during this stage, the rumen is not fully developed, digestive capacity remains limited, and the immune system is still immature. As passive immunity conferred by colostrum gradually declines, calves exhibit reduced disease resistance and stress tolerance, rendering them susceptible to health challenges such as diarrhoea, especially when exposed to stressors like dietary transitions and environmental fluctuations. These factors can substantially impair growth and farming efficiency (31–33).

Oxidative stress is a key contributor to compromised calf health. Excess reactive oxygen species (ROS) can target polyunsaturated fatty acids in cell membranes, resulting in lipid peroxidation, cellular dysfunction, and subsequent digestive disorders and diarrhoea (34). In this study, serum antioxidant markers including total antioxidant capacity (T-AOC), total superoxide dismutase (T-SOD), and glutathione peroxidase (GSH-Px) were measured to assess the body’s oxidative defense. Malondialdehyde (MDA), a byproduct of lipid peroxidation, served as an indicator of oxidative damage (35, 36). Humoral immunity was evaluated through immunoglobulin levels (IgG, IgA, and IgM), while non-specific immunity was assessed via lysozyme (LZM) and acid phosphatase (ACP) activity. Cellular immunity was reflected by cytokine levels (IL-2, IL-10, IL-6 and TNF-*α*) and lymphocyte proliferation capacity (LPC), all of which play vital roles in maintaining immune homeostasis and defending against infections (37–40).

Yeast culture (YC), a functional feed additive, is rich in *β*-glucans, mannans, organic acids, bioactive amino acids, vitamins, and various enzymes. It has been demonstrated to regulate gut microbiota composition, improve nutrient digestibility, enhance immune function, and boost antioxidant capacity (23, 41, 42). Previous studies by our team have shown that YC supplementation improves serum antioxidant markers (T-AOC, T-SOD, GSH-Px) and elevates immunoglobulin concentrations (IgG, IgA, IgM), as well as immune effectors such as LZM, and IL-1β in beef cattle, thereby enhancing their antioxidant and immune responses (12). In dairy cows, YC was found to significantly increase LZM levels, ACP activity, lymphocyte proliferation, and neutrophil phagocytic capacity, supporting improvements in innate immunity (43). These findings provide a theoretical foundation for the application of YC in calf nutrition.

In this study, supplementation with YC significantly increased the activity of T-SOD and GSH-Px, and elevated T-AOC levels in calves, while reducing serum MDA concentrations, indicating an enhanced antioxidant defense system and mitigation of oxidative stress. Additionally, the YC group exhibited significantly higher serum levels of IgG, IgA, IgM, LZM, ACP, IL-2, IL-10, and improved LPC, IL-6 levels were significantly reduced, suggesting that YC enhances both humoral and cellular immune functions. Most notably, the diarrhoea incidence was significantly lower in the YC group. This improvement is likely due to the combined effects of YC on intestinal microflora modulation, enhanced mucosal immunity, and reduced inflammation. The underlying mechanisms may involve *β*-glucans and mannans binding to intestinal receptors such as Toll-like receptors, thereby activating antigen-presenting cells (e.g., macrophages, dendritic cells), and stimulating the secretion of immune-regulatory cytokines like IL-2 and IL-10, which enhance immune responsiveness (44, 45). Furthermore, yeast-derived organic acids, amino acids, and vitamins may optimize intestinal pH, suppress pathogenic bacterial growth, reduce adhesion of harmful microorganisms, and ultimately lower the risk of diarrhoea (46). The antioxidant bioactives in YC also neutralize excess ROS, alleviate oxidative stress, and protect the intestinal mucosal barrier, promoting gut integrity and health (47).

Taken together, the results of this study and previous domestic and international research suggest that YC supplementation significantly reduces diarrhoea incidence in lactating calves by strengthening antioxidant defenses, enhancing immune function, and maintaining intestinal health. These benefits contribute to improved growth performance and support the use of YC as a safe, eco-friendly, and effective nutritional strategy in modern calf rearing systems.

### 4.3 Effects of yeast culture on intestinal microbiota structure and KEGG functional profiles in lactating calves

In recent years, metagenomic sequencing has emerged as a powerful tool for investigating the gut microbiota of animals. By enabling high-throughput sequencing of microbial community DNA, it allows for comprehensive identification of microbial taxa and community structures, while also providing insights into the distribution of functional genes and metabolic potential. This approach offers strong evidence for elucidating the mechanisms by which feed additives influence host health (48, 49). In calf studies specifically, metagenomics has been widely employed to assess how dietary interventions, environmental stressors, and disease states modulate intestinal microbial communities.

To evaluate microbial community structure, alpha diversity metrics are commonly used. The Chao index reflects species richness, i.e., the total number of microbial species in a given sample. The Shannon index accounts for both species richness and evenness, serving as a comprehensive indicator of microbial diversity. The Simpson index measures the dominance of certain species within a community, with lower values indicating higher diversity (50). In the present study, the Chao index was significantly higher in the yeast culture (YC) group compared to the control group (*p* < 0.05), while the Shannon and Simpson indices showed an increasing and decreasing trend, respectively, though not statistically significant (*p* > 0.05). These results suggest that yeast culture supplementation enhances microbial richness and potentially improves microbial diversity in the intestines of calves. These findings are consistent with previous studies by Rostoll Cangiano et al. (2) and Liu et al. (51), which also reported that yeast cultures improve gut microbial diversity in livestock.

At the phylum level, *Bacillota* (formerly *Firmicutes*) and *Bacteroidota* (formerly *Bacteroidetes*) were the dominant phyla observed in both the control and YC groups. This aligns with findings from Suhinin et al. (52), who reported that these two phyla constitute more than 80% of the intestinal microbiota in healthy calves. *Bacillota* includes important genera such as *Ruminococcus* and *Lactobacillus*, which contribute to fiber and starch degradation, production of short-chain fatty acids (SCFAs), and maintenance of intestinal mucosal health (53). *Bacteroidota* is known for its ability to degrade complex polysaccharides and plays a crucial role in maintaining intestinal nutrient balance and regulating immune function (54).

Further analysis revealed that the relative abundance of *Phocaeicola plebeius*, *Ruminococcus* sp., *Segatella copri* (formerly *Prevotella copri*), and *Candidatus Scatovivens faecipullorum* was significantly increased in the YC group, while the abundance of *Candidatus Cryptobacteroides* sp. and *Dorea* sp. was significantly reduced. Previous studies have shown that *Phocaeicola plebeius* possesses strong polysaccharide-degrading capabilities and contributes to mucosal repair and immune modulation through SCFA production (55). *Ruminococcus* species are key fiber degraders, known to produce butyrate, maintain intestinal barrier integrity, and exert anti-inflammatory effects (56). Recent studies have highlighted the important roles of *Segatella copri* in immune regulation and gut colonization (57, 58). Although limited research is available on *Candidatus Scatovivens faecipullorum*, preliminary evidence suggests its potential role in fiber degradation and intestinal health (59). Conversely, *Candidatus Cryptobacteroides* sp. has been linked to chronic inflammation and metabolic disorders (60), while *Dorea* sp. has been associated with elevated host inflammation and an increased risk of diarrhoea (61–63).

Spearman correlation analysis in this study revealed that serum total antioxidant capacity (T-AOC) was significantly positively correlated with the abundance of *Phocaeicola plebeius* and *Segatella copri* (*p* < 0.01), suggesting that these bacteria may enhance antioxidant defense through SCFA production or secretion of antioxidant metabolites. Malondialdehyde (MDA) levels were positively correlated with *Candidatus Cryptobacteroides* sp. and negatively correlated with *Ruminococcus* sp., *Segatella copri*, and *Candidatus Scatovivens faecipullorum* (*p* < 0.01), indicating that an increase in beneficial bacterial populations may help mitigate oxidative stress. Further analysis revealed that the relative abundances of *Phocaeicola plebeius*, *Ruminococcus* sp., *Segatella copri*, and *Candidatus Scatovivens faecipullorum* were positively associated with serum immune function markers, including IgG, IgA, IgM, LZM, ACP, IL-2, IL-10, and LPC, while showing varying degrees of negative association with TNF-*α* and IL-6. In contrast, the abundances of *Candidatus Cryptobacteroides* sp. and *Dorea* sp. exhibited positive correlations with TNF-α and IL-6, and negative correlations with the other immune function indicators to varying extents. These findings suggest that yeast culture enhances immune function and antioxidant capacity by promoting beneficial microbiota and suppressing potentially harmful taxa, thereby reducing the incidence of diarrhoea.

This study further revealed that dietary supplementation with YC during lactation significantly modulated immune signalling pathways associated with the intestinal microbiota of calves. KEGG functional enrichment analysis showed that the T cell receptor (TCR) and B cell receptor (BCR) signalling pathways were markedly upregulated in the YC group, with PAK1 and ERK (MAPK1/3) in the TCR pathway, and ERK (MAPK1/3) in the BCR pathway, exhibiting significantly increased expression. PAK1 and ERK1/2 (MAPK1/3) are key nodes in the TCR/BCR signalling cascade, mediating processes such as immune synapse formation, cytoskeletal remodelling, transcription factor activation, and lymphocyte proliferation and survival (64–66). These findings suggest that YC may enhance host adaptive immune responses by amplifying T/B lymphocyte signal transduction, thereby promoting their activation, proliferation, and effector functions. Furthermore, correlation analysis indicated that genes enriched in the TCR and BCR signalling pathways were strongly associated with serum antioxidant markers and immune function indicators. This supports the hypothesis that YC may influence host immune signalling pathways by modulating the gut microbiota and its metabolites (e.g., short-chain fatty acids) (23, 67, 68), consequently improving antioxidant capacity and immune function, and ultimately safeguarding calf health.

In summary, supplementation with YC during the suckling period enhanced intestinal microbial richness and diversity, promoted the proliferation of beneficial taxa such as *Phocaeicola plebeius* and *Ruminococcus* spp., and inhibited the growth of potentially harmful bacteria such as *Candidatus Cryptobacteroides* spp. By regulating the intestinal microbiome and its metabolic outputs, YC activated T/B cell receptor signalling pathways, upregulated core signalling genes such as PAK1 and ERK (MAPK1/3), and strengthened immune responses and antioxidant capacity. Collectively, these effects contributed to improved intestinal health, reduced diarrhoea incidence during the suckling period, and enhanced growth performance, providing a solid microbiological basis for ruminant health management (23, 64, 69).

This study investigated the effects of YC on calf growth performance, diarrhoea incidence, antioxidant capacity, immune function, and gut microbiota composition at a macro level. However, mechanistic insights remain limited, and addressing this gap is the focus of our ongoing research. Emerging approaches may offer novel perspectives, such as transcriptomic and epitopic spatial co-indexed sequencing (CITE), spatial ATAC-RNA-protein (DBiT-ARP) sequencing, and Perturb-DBiT. These advanced techniques could help elucidate the role of YC in driving gastrointestinal growth and development, define the structural composition of microbial communities, and reveal the developmental and activation mechanisms of immune tissues under both physiological and pathological conditions (70–72). At the same time, although the present study was limited to the preweaning period, early-life microbial and immune modulation may exert long-lasting effects. Previous studies have demonstrated that interventions during early development can influence the maturation trajectory of the gut microbiota and the education of the immune system (2, 50, 73). Therefore, future investigations tracking calves into the post-weaning period are warranted to determine whether the beneficial effects of yeast culture are sustained.

## 5 Conclusion

This study systematically evaluated the effects of yeast culture supplementation on the growth performance, health indicators, and intestinal microbiota composition of lactating Holstein calves. The results demonstrated that yeast culture significantly enhanced average daily feed intake and daily weight gain, reduced the incidence of diarrhoea, and improved overall growth performance. Furthermore, yeast culture supplementation notably increased antioxidant capacity, as evidenced by elevated serum levels of total superoxide dismutase (T-SOD), total antioxidant capacity (T-AOC), and glutathione peroxidase (GSH-Px), while concurrently reducing malondialdehyde (MDA) levels, indicating effective mitigation of oxidative stress. Additionally, significant increases were observed in the serum levels of immunoglobulins (IgG, IgA, IgM), lysozyme (LZM), acid phosphatase (ACP), and various cytokines, reduced IL-6 and TNF-*α* levels, suggesting that yeast culture supplementation enhanced both humoral and cellular immune functions. Metagenomic sequencing analysis revealed that yeast culture positively influenced the intestinal microbiota structure, increasing the abundance of beneficial bacteria, such as *Phocaeicola plebeius*, *Ruminococcus* sp., *Segatella copri*, and *Candidatus Scatovivens faecipullorum*, while inhibiting the growth of potentially harmful bacteria, such as *Candidatus Cryptobacteroides* sp. and *Dorea* sp. Correlation analysis further showed that the proliferation of beneficial bacteria was closely associated with improvements in antioxidant capacity, immune function, and a reduction in diarrhoea incidence in calves. Further functional pathway analysis suggests that YC may enhance immune responses and antioxidant capacity, improve gut health, reduce diarrhoea during lactation, and promote growth performance by modulating host immune signalling networks via metabolites produced by beneficial bacteria, thereby activating T cell receptor and B cell receptor signalling pathways.

Overall, yeast culture supplementation significantly improved both the health status and growth performance of lactating Holstein calves. This was achieved through a multidimensional regulation of the immune system, antioxidant system, and intestinal microbiota, highlighting the promising potential of yeast cultures for early-stage nutritional and health management in calves.

## Data Availability

The datasets presented in this study can be found in online repositories. The names of the repository/repositories and accession number(s) can be found in the article/Supplementary material.

## Appendix

**Table A1:** Fermentation substrate composition.

Item	Content
Wheat bran (%)	15
Spraying corn bran (%)	12
Corn (%)	16
Rice bran (%)	10
DDGS (%)	8
Corn germ meal (%)	27
Soybean meal (%)	12

**Table A2:** Nutritional components of yeast culture.

Item	Yeast culture
Dry matter (%)	≥88
Crude protein (%)	≥20
Crude ash (%)	≤9
Neutral detergent fiber (%)	≤34
Acid detergent fiber (%)	≤20
Live yeast cells (cfu/g)	≥10^6^
β-glucan (%)	≥0.5
mannan (%)	≥0.5
