# Supplementary material for: Yeast culture improves growth, antioxidant status, immunity, and gut microbiota homeostasis in preweaning Holstein calves
Source: Front Vet Sci. 2025 Sep 8;12:1670912. doi: 10.3389/fvets.2025.1670912 (PMC12452099; doi:10.3389/fvets.2025.1670912)
Supplement: Supplementary file 2 [file Table_1.docx]

Appendix 1 Fermentation substrate composition.

| Item | Content |
| --- | --- |
| Wheat bran (%) | 15 |
| Spraying corn bran (%) | 12 |
| Corn (%) | 16 |
| Rice bran (%) | 10 |
| DDGS (%) | 8 |
| Corn germ meal (%) | 27 |
| Soybean meal (%) | 12 |

Appendix 2 Nutritional components of yeast culture.

| Item | Yeast culture |
| --- | --- |
| Dry matter (%) | ≥88 |
| Crude protein (%) | ≥20 |
| Crude ash (%) | ≤9 |
| Neutral detergent fiber (%) | ≤34 |
| Acid detergent fiber (%) | ≤20 |
| Live yeast cells (cfu/g) | ≥10^6^ |
| β-glucan (%) | ≥0.5 |
| mannan (%) | ≥0.5 |
